# Immobilzation‐Induced Muscle Atrophy Exacerbates Vascular Calcification and Abdominal Aortic Aneurysms Through Irisin Downregulation

**DOI:** 10.1111/ggi.70865

**Published:** 2026-10-15

**Authors:** Zehan Song, Bo‐Kyung Son, Miya Oura, Michiko Nanao‐Hamai, Koichi Toyoshima, Aya Komuro, Sumito Ogawa, Masahiro Akishita

**Affiliations:** ^1^ Department of Geriatric Medicine, Graduate School of Medicine The University of Tokyo Tokyo Japan; ^2^ Coproduction of Inclusion, Diversity and Equity (IncluDE) Center The University of Tokyo Tokyo Japan; ^3^ Tokyo Metropolitan Institute for Geriatrics and Gerontology Tokyo Japan

**Keywords:** abdominal aortic aneurysm, inflammation, irisin, muscle atrophy, vascular calcification

## Abstract

**Background:**

Sarcopenia is widely recognized that affects multiple organs, including the cardiovascular system. Clinical studies have consistently shown associations between sarcopenia and vascular aging phenotypes such as vascular calcification, atherosclerosis, and abdominal aortic aneurysms (AAA). However, the molecular mechanisms by which muscle deterioration contributes to vascular degeneration remain poorly understood.

**Methods:**

To elucidate muscle–vascular crosstalk, we established a murine model combining inflammation‐associated muscle atrophy induced by hindlimb immobilization with CaPO₄‐induced AAA. Vascular degeneration was evaluated by Elastica van Gieson staining for elastic fiber integrity and von Kossa staining for calcification. The role of the muscle‐derived myokine irisin was examined through recombinant irisin administration and pharmacological inhibition using the αVβ5 integrin antagonist cilengitide.

**Results:**

Muscle atrophy markedly accelerated vascular calcification and AAA formation in the combined model compared with AAA alone. This was accompanied by enhanced osteogenic phenotype, downregulation of the growth arrest‐specific gene 6 (Gas6)/pAkt survival pathways, and pronounced inflammatory responses in the aortic wall. We found that muscle atrophy significantly reduced muscular and circulating irisin levels. Irisin administration attenuated vascular calcification and AAA progression, whereas irisin signaling inhibition exacerbated these vascular pathologies.

**Conclusions:**

This study provides experimental evidence that muscle atrophy directly accelerates vascular calcification and AAA formation. We also identify irisin as a key molecular mediator linking muscle atrophy to vascular degeneration, highlighting muscle‐derived signaling as a critical target for preventing vascular pathologies.

## Graphical abstract

Immobilization‐induced muscle atrophy accelerates vascular calcification and abdominal aortic aneurysm (AAA) formation by suppressing irisin, a key mediator with vascular anti‐inflammatory effects in male mice. Our combined murine model reveals a mechanistic link between muscle atrophy and vascular disease, highlighting irisin signaling as a potential therapeutic target for suppressing vascular inflammation.

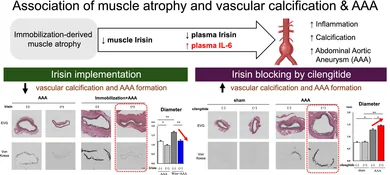

## 1 Introduction

Recently, accumulating evidence indicates that declines in skeletal muscle mass and strength are associated with a wide range of age‐related diseases, particularly cardiovascular disorders [1]. In detail, individuals with sarcopenia exhibit a higher prevalence of vascular calcification, a hallmark of vascular aging [2], as well as vascular diseases such as atherosclerosis and abdominal aortic aneurysm (AAA) [3, 4]. These observations suggest a close link between muscle deterioration and vascular aging/diseases. However, the molecular mechanisms remain poorly understood.

Chronic inflammation has been proposed as a common biological pathway underlying both muscle deterioration and vascular aging [5]. Especially regarding muscle‐derived impacts, myokines have emerged as important regulators of inflammatory processes. Among them, irisin, a peptide cleaved from fibronectin type III domain‐containing protein 5 (FNDC5), exerts anti‐inflammatory and anti‐atherosclerotic effects [6, 7]. Furthermore, circulating irisin levels decline with aging and physical inactivity, raising the possibility that irisin serves as a key regulator linking skeletal muscle and vessels [8, 9]. However, direct experimental evidence elucidating the causal impact of muscle atrophy on vascular pathology is limited, in part due to the lack of appropriate animal models that simultaneously capture muscle degeneration and vascular pathology under inflammatory conditions.

In the present study, using a combined murine model of muscle atrophy and AAA, we investigated the impact of muscle atrophy on vascular disease progression [10, 11]. Furthermore, we sought to identify molecular mediators linking skeletal muscle deterioration to vascular pathology with a particular focus on the role of irisin.

## 2 Materials and Methods

### 2.1 Animals

Ten‐week‐old male C57BL/6J mice (Sankyo Labo Service Corporation, Tokyo, Japan) housed under controlled conditions (12‐h light/dark cycle, 21°C ± 3°C, humidity 40% ± 10%) with free access to water and standard chow. All surgical procedures and samples (plasma, skeletal muscle, and aorta) collection were performed under anesthesia induced by intraperitoneal injection of a mixture of medetomidine hydrochloride (0.3 mg/kg; Kyoritsu Seiyaku, Tokyo, Japan), midazolam (4 mg/kg; Sandoz Pharma K.K., Tokyo, Japan), and butorphanol (5 mg/kg; Meiji Seika Pharma, Tokyo, Japan) at a dose of 0.05 mL per 10 g of body weight, administered once prior to each procedure.

All animal experiments were approved by the Animal Experiment Committee of the University of Tokyo (approval id: Medical‐P20‐009) and conducted in accordance with the NIH Guide for the Care and Use of Laboratory Animals.

### 2.2 Hindlimb Immobilization Model

Mouse hindlimbs were wrapped with surgical tape and aluminum wire (Daidohant Co. Ltd., Osaka, Japan) was used to fix both sides, in accordance with the protocol outlined in a previous study [10]. For evaluation, gastrocnemius muscles weight measured and grip strength was assessed using a grip strength meter (MK‐380CM/R; Muromachi Kikai, Tokyo, Japan). The wire‐hang test was conducted using an inverted wire mesh in a 20‐cm‐high acrylic chamber.

### 2.3 Abdominal Aortic Aneurysm (AAA) Model

AAA was induced by periaortic application of 0.5 M CaCl_2_ (Wako, Osaka, Japan), followed by phosphate‐buffered saline (PBS; Wako), to the infrarenal abdominal aorta [11]. Sham‐operated mice underwent laparotomy without CaCl_2_ application. After 42 days, harvested aortas were photographed digitally, and maximal aortic diameters were measured at the most dilated region. For RNA and protein analyses, due to limited tissue quantity, two aortas were pooled, particularly in the sham group.

### 2.4 Histological Staining and Immunohistochemistry

Aortas were fixed in 4% paraformaldehyde, dehydrated, embedded in paraffin, and sectioned into serial 5 μm‐thick sections. Elastic fibers were visualized using Elastica van Gieson (EVG) staining. Vascular calcification was assessed by calcium deposition using Von Kossa staining without nuclear counterstaining. Calcified areas were quantified as the ratio of Von Kossa–positive area to tunica media area, defined based on adjacent EVG‐stained sections.

To evaluate macrophage infiltration in AAA lesion, paraffin‐embedded sections were incubated overnight with anti‐CD68 antibody (#MCA1957GA; Bio‐Rad, USA). Nuclei were counterstained with DAPI (Sigma‐Aldrich).

### 2.5 RNA Isolation and Quantitative Real‐Time PCR

Total RNA was extracted using RNeasy Mini Kits (#74106; Qiagen, Hilden, Germany) and cDNA was synthesized using Omniscript RT Kits (#205110; Qiagen) and oligo(dT) primers (Invitrogen, USA). Quantitative real‐time PCR was performed using Fast SYBR Green Master Mix on a StepOnePlus system (Applied Biosystems, USA). Gene expression levels were normalized to GAPDH. Primer sequences are listed in Table S1.

### 2.6 Western Blot Analysis

Protein samples were separated by SDS‐PAGE and transferred to PVDF membranes (Fluoro Trans W; Pall Corp., Japan). Membranes were incubated overnight with primary antibodies against Gas6 (#A8545), pAkt (#9271), Akt (#9272), irisin (FNDC5; #ab174833), β‐actin (#A5441), and β‐tubulin (#T4026). The corresponding horseradish peroxidase–conjugated secondary antibodies (GE Healthcare) were incubated. Signals were detected using ECL Prime (GE Healthcare) and images were acquired using a LuminoGraph I system (ATTO, Japan).

### 2.7 Irisin Administration and Inhibition

Recombinant irisin (0.21 μg/g; Peprotech, USA) or PBS was administered intraperitoneally every 3 days throughout the 56‐day experimental period. For irisin inhibition, mice received intraperitoneal injections of cilengitide (4 μg/g; Selleck Chemicals, TX, USA), an integrin αVβ3/αVβ5 inhibitor, every other day for 42 days after AAA induction. Control mice received PBS injections.

### 2.8 Plasma IL‐6 and Irisin Measurements

Plasma IL‐6 levels were measured using Quantikine ELISA kits for mouse (M6000B‐1) (R&D Systems, USA). Plasma irisin concentrations were determined using a competitive ELISA kit (AG‐45A‐0046YEK‐KI01; Adipogen, USA) for mouse according to the manufacturers' protocols.

### 2.9 Statistical Analysis

Statistical analyses were performed using GraphPad Prism version 6. Comparisons among multiple groups were conducted using one‐way ANOVA followed by Tukey's post hoc tests. Two‐group comparisons were performed using Student's *t*‐test or Mann–Whitney *U* test, as appropriate. Normality was assessed using the Shapiro–Wilk test. Correlation analysis was performed using Spearman's rank correlation coefficient. Data are expressed as mean ± SEM, and *p* < 0.05 was considered statistically significant.

## 3 Results

### 3.1 Immobilization‐Induced Muscle Atrophy Exacerbates Vascular Calcification and AAA Formation

Compared with mice subjected to AAA alone, immobilized mice significantly exacerbated AAA formation, with increased maximal aortic diameters below the renal artery bifurcation (Figure 1A,C). Elastica van Gieson (EVG) staining revealed elastic fibers in the tunica media were markedly destroyed in immobilized mice with AAA compared with AAA‐only mice (Figure 1B). Furthermore, Von Kossa staining demonstrated extensive aortic calcification in immobilized mice with AAA, which was significantly greater than in mice with AAA alone (Figure 1B,D).

**FIGURE 1:**
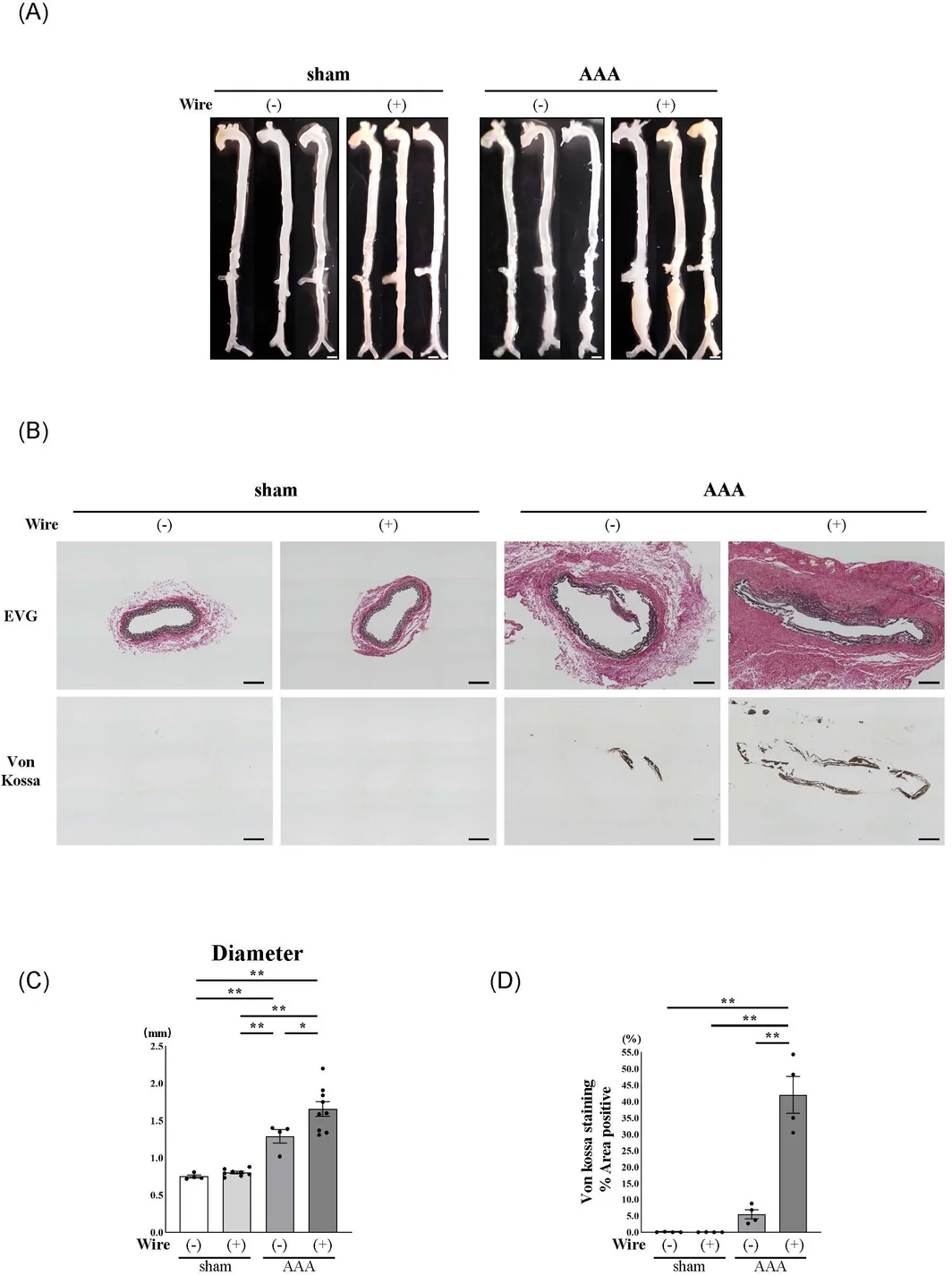
Immobilization accelerates vascular calcification and AAA formation. (A) Representative aorta and (B) Elastica van Gieson (upper panel) and Von Kossa. (lower panel) staining in the representative aorta of AAA and sham with or without immobilization (Wire) mice. (C) Aortic diameter (Each black dot represents an individual data point.) and (D) Von Kossa‐positive area in AAA and sham with or without immobilization (Wire) mice (Each black dot represents an individual data point.). Data are shown as means ± SEM. Statistical analysis was performed using one‐way ANOVA followed by Tukey's post hoc test. *p < 0.05, **p < 0.01. Aortic diameter measurements (A and C), *n* = 4, 8, 4, and 9 mice per group, and other analyses (B and D), *n* = 4 per group. Scale bars: *A* = 1 mm; *B* = 100 μm. AAA, abdominal aortic aneurysm; SEM, standard error of the mean.

### 3.2 Effects of Immobilization on Molecular Mechanisms of Vascular Calcification

Vascular calcification has two well‐established pathological mechanisms: [1] The phenotype shift from VSMCs to osteoblasts by the upregulation of runt‐related transcription factor 2 (Runx2), a key transcription factor [12] and [2] apoptosismediated by the downregulation of the growth arrest‐specific gene 6 (Gas6)/phosphorylated Akt (pAkt) survival pathway [13]. In this study, we found that aortic mRNA expression of Runx2 was significantly increased in immobilized mice with AAA compared with AAA‐only mice (Figure 2A). In contrast, protein expression levels of Gas6 and phosphorylated Akt (pAkt) were significantly reduced in the aorta of immobilized mice with AAA (Figure 2B–D).

**FIGURE 2:**
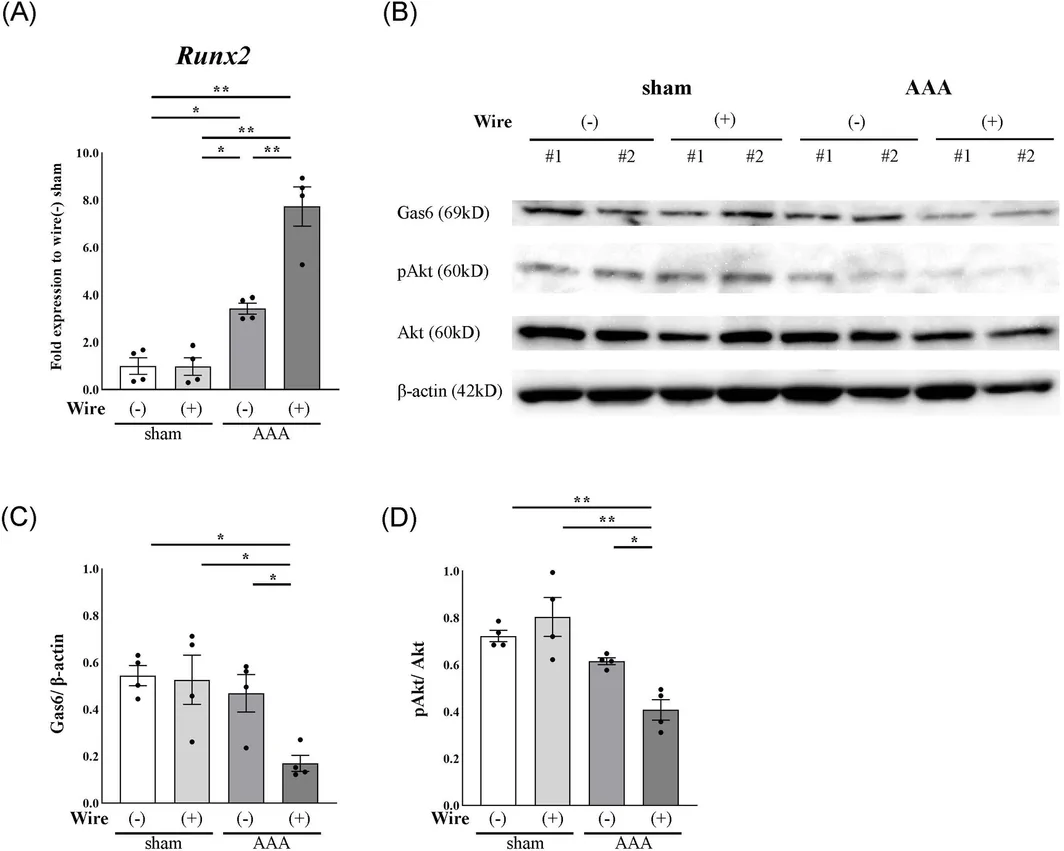
Effect of immobilization on osteoblastic transition and apoptosis in AAA lesions. (A) Aortic Runx2 mRNA expression (Each black dot represents an individual data point.) and (B) Representative western blots of apoptosis‐related proteins Gas6, pAkt, Akt, and β‐actin in the aorta of AAA and sham with or without immobilization (Wire) mice. Quantitative analysis of Gas6 (C) and pAkt (D) expression. Each black dot of (C) and (D) represents an individual data point. Data are shown as means ± SEM. Statistical analysis was performed using one‐way ANOVA followed by Tukey's post hoc test. *p < 0.05, **p < 0.01. *n* = 4 per group. AAA, abdominal aortic aneurysm; Akt, protein kinase B; Gas6, growth arrest‐specific 6; pAkt, phosphorylated protein kinase B; SEM, standard error of the mean.

### 3.3 Immobilization Promotes Inflammatory Responses in AAA Lesions

Increased infiltration of CD68‐postivie macrophage was shown in pronounced AAA lesions of immobilized mice with AAA compared with AAA alone (Figure 3A,B). Consistently, mRNA expression of inflammatory cytokines, including IL‐1β and IL‐6 was significantly upregulated in immobilized mice with AAA (Figure 3C). We also found that plasma IL‐6 concentrations significantly elevated in immobilized mice with AAA compared with AAA alone (Figure 3D).

**FIGURE 3:**
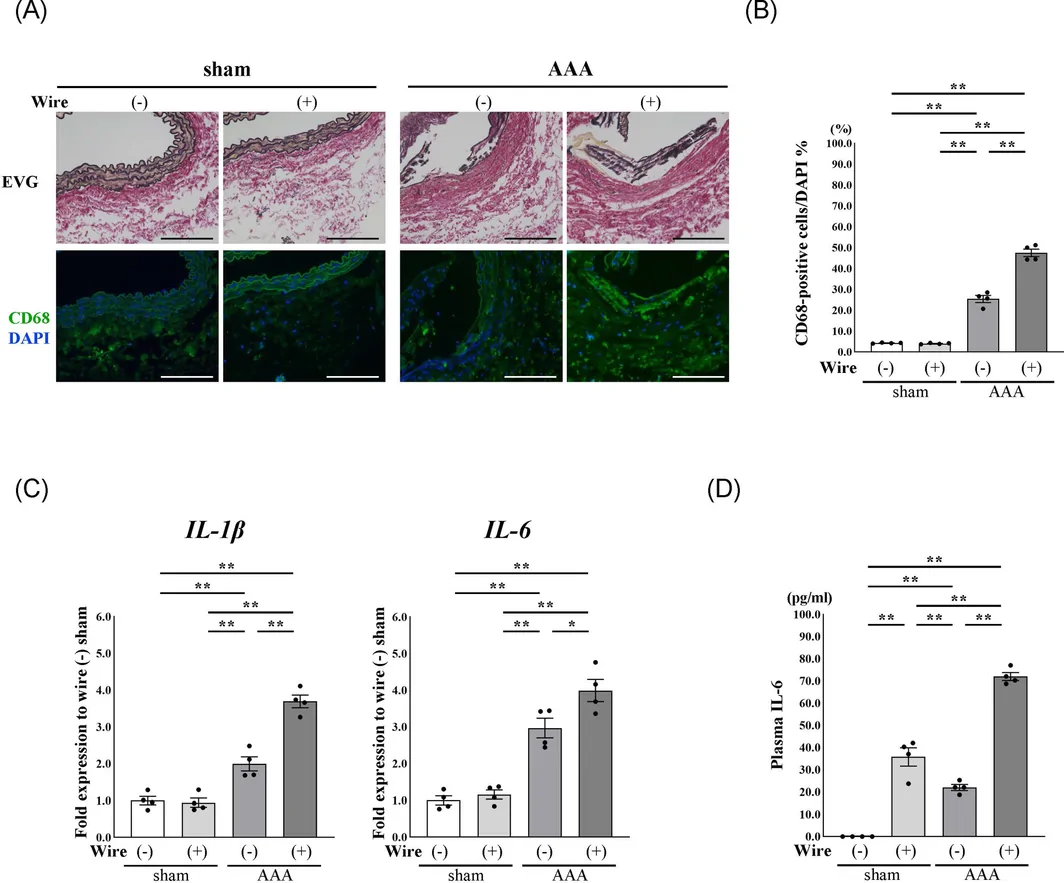
Effect of immobilization on macrophage infiltration and inflammatory responses in AAA lesions. (A) Elastica van Gieson staining (upper panel) and immunostaining of CD68‐positive macrophage and DAPI (lower panel) in the representative aorta of AAA and sham with or without immobilization (Wire) mice. (B) Quantitative analysis of CD68‐positive macrophage. (C) Aortic mRNA expression of IL‐1β and IL‐6. (D) Enzyme‐linked immunosorbent assays of plasma IL‐6 in mice. Each black dot of (B‐D) represents an individual data point. Data are shown as means ± SEM. Statistical analysis was performed using one‐way ANOVA followed by Tukey's post hoc test. *p < 0.05, **p < 0.01. *n* = 4 per group. Scale bar = 100 μm. AAA, abdominal aortic aneurysm; IL‐6, interleukin‐6; SEM, standard error of the mean.

### 3.4 Irisin Administration Attenuates Vascular Calcification and AAA Formation Exacerbated by Immobilization

In skeletal muscle, protein expressions of irisin declined significantly in a time‐ dependent manner during hindlimb immobilization (Figure 4A). Plasma irisin concentrations also progressively decreased over 28 days of immobilization (Figure S1).

**FIGURE 4:**
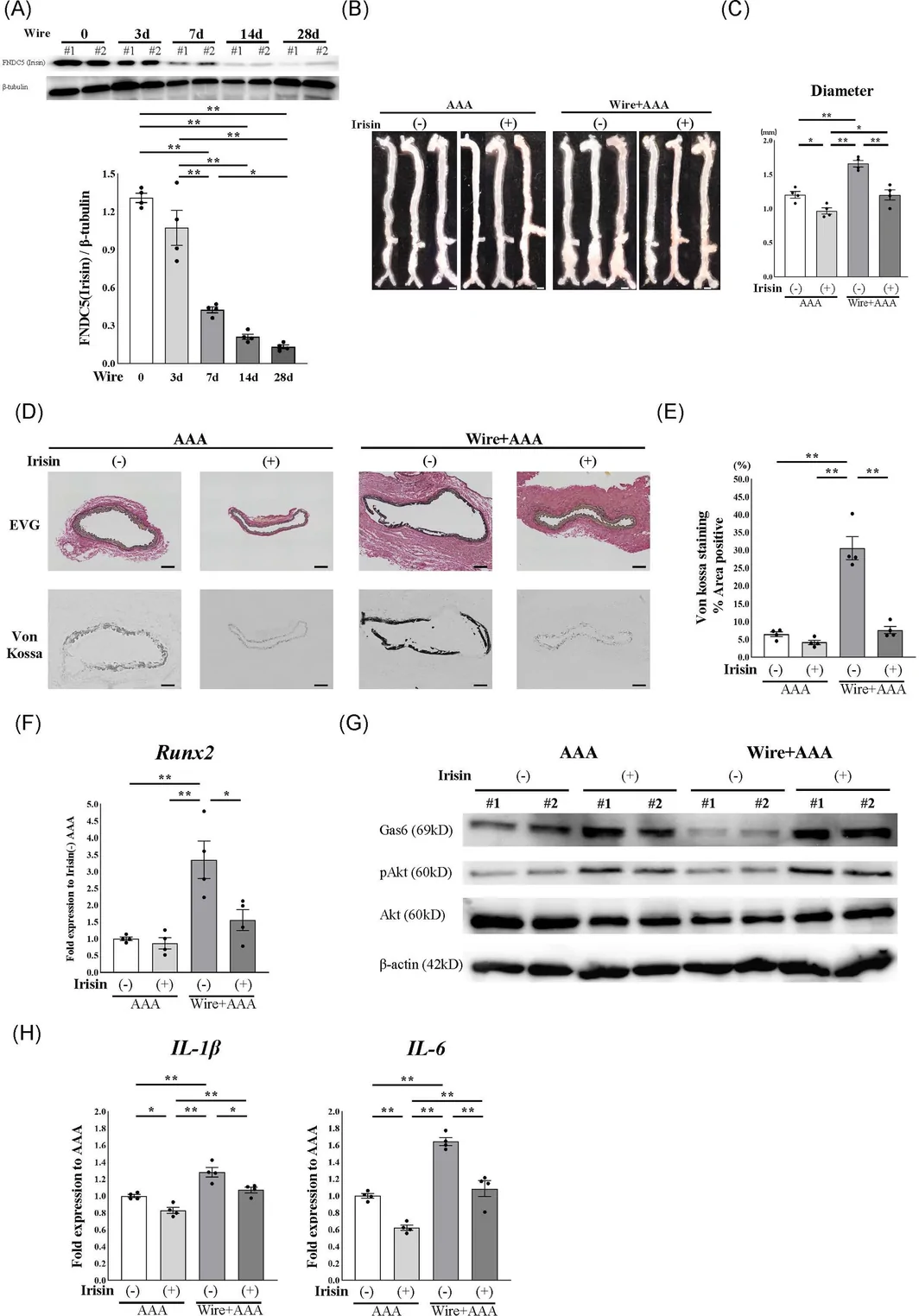
Effect of recombinant irisin on vascular calcification and AAA formation exacerbated by immobilization. (A) Representative western blots of FNDC5 (irisin) in skeletal muscle of mice and quantitative analysis of FNDC5 (irisin). (B) Representative aorta and (C) quantitative analysis of aortic diameter with or without irisin administration in AAA and AAA with immobilization (Wire+AAA) mice. (D) Elastica van Gieson (upper panel) and Von Kossa (lower panel) staining of the representative aorta with or without irisin administration in AAA and AAA with immobilization (Wire+AAA) mice. (E) Quantitative analysis of Von Kossa staining. (F) Aortic Runx2 mRNA expression and (G) representative western blots of apoptosis‐related proteins Gas6, pAkt, Akt, and β‐actin in the aorta with or without irisin administration in AAA and AAA with immobilization (Wire+AAA) mice. (H) Aortic mRNA expression of IL‐1β and IL‐6. Each black dot of (A), (C), (E), (F) and (H) represents an individual data point. Statistical analysis was performed using one‐way ANOVA followed by Tukey's post hoc test. *p < 0.05, **p < 0.01 (*n* = 4 per group). Scale bars: *A* = 1 mm; *B* = 100 μm. AAA, abdominal aortic aneurysm; SEM, standard error of the mean.

Recombinant irisin was administered during immobilization and AAA induction. As shown in Figure 4B,C, irisin administration significantly reduced maximal aortic diameters in mice with AAA, both with and without immobilization. Von Kossa staining also revealed a marked reduction of calcium deposition in the aorta of immobilized mice with AAA by irisin administration (Figure 4D,E).

Irisin administration significantly inhibited Runx2 expression in the aorta of immobilized mice with AAA (Figure 4F). Conversely, Gas6 and pAkt expression levels were significantly increased following irisin administration (Figure 4G). Regarding inflammatory responses, aortic mRNA expression of IL‐1β and IL‐6 (Figure 4H) was significantly suppressed by irisin administration. Furthermore, irisin administration significantly reduced plasma IL‐6 levels in immobilized mice with AAA (Figure S2).

### 3.5 Blockade of Irisin Signaling Aggravates Vascular Calcification and AAA Formation

In addition, cilengitide was used to explore the direct involvement of the integrin αVβ5 pathway, a characterized receptor axis for irisin, in the AAA local environment, rather than claiming a direct casual blockade of immobilization‐induced endogenous irisin. As expected, cilengitide significantly increased maximal aortic diameter and aortic calcification compared with control‐treated AAA mice (Figure 5A–D).

**FIGURE 5:**
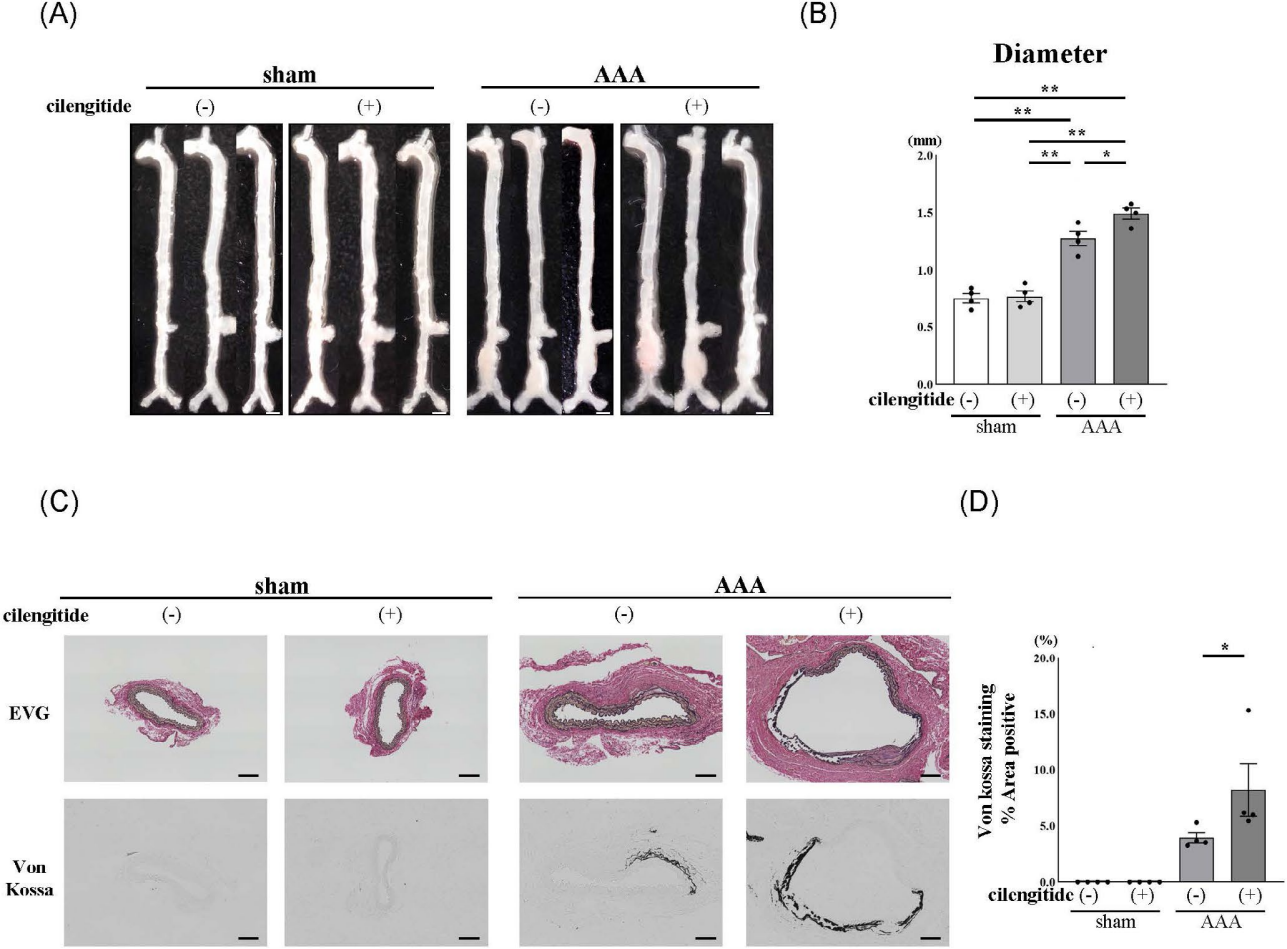
Effect of cilengitide on vascular calcification and AAA formation. (A) Representative aorta and (B) quantitative analysis of aortic diameter with or without cilengitide, an integrin αVβ5 inhibitor administration in AAA or sham mice. Each black dot represents an individual data point. (C) Elastica van Gieson (upper panel) and Von Kossa (lower panel) staining of the representative aorta with or without cilengitide administration in AAA or sham mice. (D) Quantitative analysis of Von Kossa staining. Each black dot represents an individual data point. Statistical analysis: (B) one‐way ANOVA followed by Tukey's post hoc test; (D) because calcification was undetected in the sham groups, the statistical comparison for Von Kossa staining was restricted to the two AAA groups using the Mann–Whitney *U* test. Data are shown as means ± SEM. *p < 0.05, **p < 0.01 (*n* = 4 per group). Scale bars: *A* = 1 mm; *B* = 100 μm. AAA, abdominal aortic aneurysm. SEM, standard error of the mean.

## 4 Discussion

In this study, we demonstrated that muscle atrophy markedly promotes vascular calcification and AAA formation using a murine model that combines immobilization and AAA induction. We found that circulating irisin levels were significantly reduced in male mice with immobilization. Moreover, we also found that irisin administration could attenuate both vascular calcification and AAA formation, whereas pharmacological inhibition of irisin signaling exacerbated these vascular pathologies. These findings suggest that irisin serves as a protective myokine that modulates vascular pathology.

Clinical evidence has indicated that low skeletal muscle mass and muscle weakness are risk factors for vascular calcification [2], which itself is closely associated with AAA progression and rupture [14]. To explore these mechanistic connections, we established a novel dual model combining immobilization‐induced muscle atrophy with CaPO_4_‐induced AAA, based on the premise that chronic inflammation represents a key shared pathogenic pathway underlying both muscle atrophy and vascular diseases [15, 16, 17]. In this study, immobilization‐induced muscle atrophy was accompanied by increased expression of inflammatory markers such as IL‐6 and F4/80, as well as upregulation of atrophic factors including MuRF1 and Atrogin‐1. The CaPO_4_‐induced AAA model manifested vascular calcification and aneurysm formation, accompanied by macrophage infiltration and inflammatory responses [10, 11]. Accordingly, this combined model offers a valuable experimental platform for investigating inflammation‐driven deterioration of both skeletal muscle and vascular tissues.

In this study, we also confirmed irisin as a key protective factor against muscle atrophy‐derived exacerbation of vascular calcification and aneurysm. These results align with several reports showing that irisin protects endothelial cells from oxidized LDL–induced apoptosis [18, 19, 20, 21] and promotes a contractile phenotype in vascular smooth muscle cells while inhibiting phenotypic switching [22]. Regarding inflammatory responses, previous studies have shown that irisin decreases inflammatory cytokines such as TNF‐α and IL‐6 and also regulates macrophage‐associated inflammation [23, 24].

Conversely, blockade of irisin signaling with cilengitide, an integrin αVβ5 inhibitor, aggravated vascular calcification and AAA formation, probably through enhanced inflammatory responses. Notably, cilengitide administration alone did not induce inflammatory responses in either the aorta or skeletal muscle (data not shown). These observations are consistent with prior evidence of integrin αVβ5‐mediated irisin signaling [25] and indicate an important anti‐inflammatory and vasculoprotective role of this pathway.

Regarding skeletal muscle, we found that irisin administration did not significantly improve muscle mass, whereas a significant improvement in muscle strength was observed in combination model mice (Figure S3). This discrepancy is considered to be that the effective concentration, delivery efficiency, and tissue‐specific sensitivity to irisin may differ between muscles and vascular tissues. Further investigation is warranted to determine dose–response relationships and tissue‐targeting properties of irisin across muscle–vascular networks.

This study has several limitations. First, experiments were conducted exclusively in young male mice, and sex‐ and age‐related differences could not be evaluated. Second, although the protective effects of irisin were evident in vivo, to clarify the cellular mechanisms underlying irisin‐mediated protection, in vitro studies using vascular smooth muscle cells or macrophages are required. Third, the immobilization model reflects secondary sarcopenia [26], and thus the roles of irisin in primary age‐related sarcopenia remain to be clarified. Furthermore, the reciprocal effects of vascular pathology on muscle atrophy were not investigated, although emerging evidence suggests bidirectional interactions between muscle and vascular systems [27]. Despite these limitations, the present study reveals a mechanistic link between immobilization‐induced muscle atrophy and vascular disease progression, and supports irisin as a potential therapeutic candidate for muscle atrophy‐associated vascular disorders.

In conclusion, the present study provides direct experimental evidence that muscle atrophy can accelerate vascular aging and disease through defined inflammatory and molecular pathways. By identifying irisin as a critical mediator of muscle–vascular crosstalk, our findings offer new mechanistic insight into how skeletal muscle deterioration contributes to vascular calcification and AAA formation.

## Funding

This study was supported by Grants‐in‐Aid for Scientific Research from the Ministry of Education, Culture, Sports, Science, and Technology of Japan (22K11697, 23H02811) and the 8th Japan Geriatrics Society (JGS) Research Grant Award in Geriatrics and Gerontology (2023).

## Ethics Statement

All animal experiments were approved by the Animal Experiment Committee of the University of Tokyo (approval id: Medical‐P20‐009) and conducted in accordance with the NIH Guide for the Care and Use of Laboratory Animals.

## Conflicts of Interest

The authors declare no conflicts of interest.

## Supporting information


**Figure S1:** Plasma irisin concentrations in immobilization male mice.
Enzyme‐linked immunosorbent assays of plasma irisin in mice. *n* = 4 per group. Statistical analysis: one‐way ANOVA followed by Tukey's post hoc test; Data are shown as means ± SEM. **p < 0.01, ^#^ *p* < 0.05 compared with all other groups. GAPDH, glyceraldehyde‐3‐phosphate dehydrogenase; SEM, standard error of the mean.

**Figure S2:** Effect of irisin administration on plasma IL‐6 levels in immobilized male mice with AAA. Plasma IL‐6 concentrations were measured by enzyme‐linked immunosorbent assay (ELISA). *n* = 4 per group. Data are presented as means ± SEM. Statistical significance was assessed by one‐way ANOVA followed by Tukey's post hoc test. **p < 0.01 versus all other groups.

**Figure S3:** Effects of immobilization, irisin administration and cilengitide administration on muscle mass (A, C, E) and grip strength (B, D, F) in mice. Data are shown as means ± SEM. Statistical analysis was performed using one‐way ANOVA followed by Tukey's post hoc test. *p < 0.05, **p < 0.01 (*n* = 4 per group).

**Table S1:** Primer Sequences used for qPCR.

## Data Availability

The data that support the findings of this study are available from the corresponding author upon reasonable request.
