# Supplementary material for: Immobilzation‐Induced Muscle Atrophy Exacerbates Vascular Calcification and Abdominal Aortic Aneurysms Through Irisin Downregulation
Source: Geriatr Gerontol Int. 2026 Oct 15;26(10):e70865. doi: 10.1111/ggi.70865 (PMC13670398; doi:10.1111/ggi.70865)
Supplement: Supplementary file 1 [file GGI-26-0-s004.pdf]

Supplementary Table 1. Primer Sequences used for qPCR

GAPDH	forward	AACTTTGGCATTGTGGAAGG
	reverse	ACACATTGGGGGTAGGAACA
Runx2	forward	AGAGTCAGATTACAGATCCCAGG
	reverse	AGGAGGGGTAAGACTGGTCATA
IL-6	forward	CAATGCTCTCCTAACAGATAAG
	reverse	AGGCATAACGCACTAGGT
TNFα	forward	CTGAACTTCGGGGTGATCGG
	reverse	GGCTTGTCACTCGAATTTTGAGA
Irisin/FNDC5	forward	AGAAGAAGGATGTGCGGATGC
	reverse	TGTTGTTATTGGGCTCGTTGTC
